# The relationship of acculturative stress with meaning in life through the mediating role of difficulties in emotion regulation and meaning-centered coping style among international students in Germany

**DOI:** 10.3389/fpsyg.2025.1639194

**Published:** 2025-08-01

**Authors:** Rasa Soufi Amlashi, Simon Forstmeier

**Affiliations:** Developmental Psychology and Clinical Psychology of the Lifespan, University of Siegen, Siegen, Germany

**Keywords:** acculturative stress, meaning in life, difficulties in emotion regulation, meaning-centered coping style, international students, Germany

## Abstract

**Introduction:**

With the increasing trend of international academic mobility, understanding the psychological outcomes of cultural transition has become crucial. The present study aimed to examine the relationship between acculturative stress and meaning in life (MIL), focusing on the mediating roles of difficulties in emotion regulation (DIER) and meaning-centered coping style (MCCS) among international students in Germany.

**Methods:**

This descriptive-correlational study recruited 443 students enrolled at German universities in 2024 through convenience sampling. Participants completed Sandhu & Asrabadi’s Acculturative Stress Scale for International Students, Gratz & Roemer’s DIER Scale, Eisenbeck et al.’s Meaning-Centered Coping Scale, and Steger et al.’s Meaning in Life Questionnaire. Data were analyzed using Pearson’s correlation coefficient and structural equation modeling (SEM) in SPSS-26 and LISREL-10.20.

**Results:**

The findings indicated that acculturative stress was directly and positively associated with the search for meaning, and indirectly associated with both the presence of meaning and the search for meaning through DIER and MCCS. Specifically, acculturative stress was positively related to DIER, which in turn was negatively associated with the presence of meaning and positively with the search for meaning. Additionally, acculturative stress was negatively related to MCCS, which was positively linked to the presence of meaning, but not significantly to the search for meaning.

**Discussion:**

These results underscore the significance of emotional regulation and MCCS in mitigating the psychological effects of acculturative stress and promoting psychological wellbeing among international students.

## Introduction

With advancements in technology and increased opportunities for mobility, educational migration has become a widespread phenomenon in the modern world. Statistical data reveal that Germany is emerging as a key destination within the global South–North student mobility framework. In 2023, approximately 485,000 international students were enrolled in higher education institutions across Germany (Gent, 2023). While this trend presents significant advantages, it also brings considerable psychological challenges for international students, affecting their overall mental health. Prior research has demonstrated that issues such as depression (Cheung et al., 2020; Gebregergis et al., 2020; Yim et al., 2023), mental health symptoms (Taušová et al., 2019), substance abuse (Paredes, 2024) and emotional distress (An et al., 2024) are prevalent among this population, highlighting the severity of the psychological risks they face.

Beyond its well-documented impact on psychological health, acculturative stress may also interfere with the process of meaning-making in international students. As individuals struggle to adapt to unfamiliar cultural environments, their capacity to construct or sustain a coherent sense of meaning can be disrupted, which may potentially compound psychological difficulties. Understanding this link is essential to clarifying how acculturative stress not only challenges mental wellbeing but may also erode fundamental psychological resources like meaning in life.

### Acculturative stress and meaning in life

Meaning in life (MIL) is a crucial factor in determining how individuals respond to major life transitions. The way people derive meaning from their lives shapes their reactions to significant events and influences the psychological outcomes of these experiences. Various theoretical frameworks have been employed to examine MIL, among which Steger’s two-factor model stands out as a prominent approach. According to Steger et al. (2006), MIL is defined as the sense made of, and significance felt regarding, the nature of one’s being and existence. This model identifies two distinct but related dimensions, namely the presence of meaning (POM) and the search for meaning (SFM). The POM refers to an individual’s perceived sense that their life is purposeful and significant, while the SFM reflects an active pursuit of deeper understanding and existential significance (Steger et al., 2006). One of the key variables that can determine the importance of MIL is acculturative stress. The challenges associated with adapting to a new cultural environment can intensify the need for meaning, either by reinforcing an existing MIL or by prompting individuals to actively seek meaning to cope with cultural transitions. Understanding these dynamics can provide valuable insights into how MIL serves as a psychological resource in the face of acculturative stress.

Acculturative stress arises when individuals encounter challenges in adapting to a new cultural environment, often stemming from unfamiliar social norms, language barriers, and differing expectations (Berry, 2006). As mentioned before, research has consistently linked acculturative stress to adverse psychological consequences, insofar as a meta-analysis by Soufi Amlashi et al. (2024) further substantiated these associations, reporting a correlation of *r* = 0.39 between acculturative stress and negative psychological outcomes and a correlation of *r* = 0.41 with depression among international students. The struggle to integrate into an unfamiliar cultural, linguistic, and social setting can generate significant emotional distress, fostering a sense of alienation and diminishing one’s ability to engage with new experiences.

However, among these challenges, MIL can play a critical role in shaping the adaptation process. Individuals who perceive a strong MIL may interpret cultural transitions as opportunities for personal growth rather than as sources of distress. Conversely, those who search for meaning may struggle to find coherence in their experiences, exacerbating the psychological burden of cultural adjustment (Pan et al., 2007). Although there is a scarcity of research to examine the relationship between acculturative stress and MIL (Pan et al., 2007, 2008) in international students, it seems that there are a number of variables capable of determining the relationship between two mentioned variables, playing a mediating role. Among these variables, two are difficulties in emotional regulation (DIER) and meaning-centered coping style (MCCS).

### Acculturative stress and MIL through DIER

Emotional regulation involves the strategies individuals employ to manage their emotional states, encompassing cognitive, behavioral, interpersonal, and intrapersonal processes (Gross and Thompson, 2007). A comprehensive understanding of emotional regulation highlights several key components of self-regulation, including emotional awareness, acceptance of emotions, goal-directed processing of distressing emotional experiences, and the adaptive use of regulation strategies in response to situational demands. Deficiencies in any of these domains indicate DIER (Gross and Thompson, 2007). Notably, impaired emotional regulation has been recognized as a significant factor contributing to the persistence of various psychological disorders. Research on international students suggests that heightened acculturative stress is closely linked to greater DIER, which in turn predisposes individuals to psychological distress, such as anxiety and depression (Mayorga et al., 2018). Furthermore, a longitudinal study conducted by Cheung et al. (2020) found that acculturative stress among international students is significantly associated with DIER. Moreover, Awasthi et al. (2022) indicated that acculturative stress in international students is significantly associated with emotion regulation strategies, such that maladaptive strategies like rumination and blaming others are linked to higher acculturative stress, whereas adaptive strategies like positive refocusing contribute to lower acculturative stress.

Additionally, a study by Baquero-Tomás et al. (2023) on university students has shown that DIER are also associated with MIL, such that lower levels of MIL are significantly related to greater DIER. According to Lin (2022), emotion regulation strategies significantly influence the MIL among college students. Specifically, cognitive reappraisal positively correlates with life meaning, while expression suppression negatively correlates with it. In summary, DIER, particularly through maladaptive strategies like expression suppression, may lead to a diminished MIL. This highlights the importance of addressing DIER as they are strongly associated with challenges in finding MIL, particularly among individuals facing emotional regulation issues.

### Acculturative stress and MIL through MCCS

MIL plays a crucial role in shaping individuals’ coping strategies, particularly in response to stress and adversity (Halama, 2014). Recent studies show that individuals with a stronger sense of meaning in life are more likely to adopt adaptive coping strategies. For example, Lin (2024) found that college students with higher MIL were more inclined to use active and constructive coping styles, which were linked to greater hope and career adaptability. Similarly, Wang et al. (2019) demonstrated that employing a meaning-focused coping technique, such as self-distanced reflection, not only enhanced positive affect but also increased participants’ post-event sense of life meaning, suggesting a reciprocal link between MIL and adaptive coping. This protective role of meaning is grounded in various theoretical frameworks, including Antonovsky’s (1987) sense of coherence model, which posits that meaningfulness is a key determinant of stress resilience. MIL provides individuals with a structured perspective that allows them to interpret stressful experiences in a way that maintains psychological stability (Park and Folkman, 1997). Empirical findings support this notion, demonstrating that individuals with a strong sense of meaning engage in coping strategies that reinforce their sense of purpose, such as benefit finding, meaning-making, and prioritization of existential values (Folkman, 2008). Moreover, longitudinal studies suggest that the use of MCCS not only alleviates distress but also enhances one’s overall MIL over time, creating a positive feedback loop (Park et al., 2008). Taken together, these findings highlight the centrality of MIL as both a foundation and an outcome of effective coping, reinforcing its role as a psychological buffer against stress (Halama, 2014).

Additionally, MCCS has been identified as a crucial strategy in managing acculturative stress, enabling people to reframe challenges in a way that fosters resilience (Halama, 2014). Research indicates that people who employ MCCS experience lower levels of stress and better psychological adjustment (Folkman, 2008; Liw, 2020). Liw (2020) found that students who engaged in MCCS reported higher life satisfaction and positive affect, even in the presence of significant acculturative stress.

### Current study

Given the increasing prevalence of international student mobility, understanding the psychological consequences of acculturative stress is considered a crucial area of research. The challenges associated with cultural adaptation can lead to significant emotional distress, necessitating the identification of protective mechanisms that facilitate students’ wellbeing. Among these mechanisms, MIL plays a pivotal role in shaping students’ capacity to navigate acculturative stress effectively. However, the pathways through which acculturative stress is associated with MIL remain underexplored, particularly concerning the roles of DIER and MCCS. Addressing this gap is essential for advancing both theoretical knowledge and practical interventions aimed at supporting international students’ psychological wellbeing. This study contributes to a growing body of literature by clarifying the indirect role of DIER and MCCS in the relationship between acculturative stress and MIL. Previous studies have established independent links between acculturative stress and DIER (Awasthi et al., 2022; Cheung et al., 2020), as well as between DIER and MIL (Baquero-Tomás et al., 2023; Liw, 2020). However, no study has directly examined DIER as a mediator between acculturative stress and MIL, highlighting a critical research gap. Similarly, while MCCS has been shown to be related to MIL by fostering adaptive coping strategies (Halama, 2014; Park et al., 2008), its role as a mediator between acculturative stress and MIL has remained relatively unexplored. Despite the well-established relationship between acculturative stress and mental health, limited research has examined how this stressor correlates with MIL through the aforementioned mediators.

**Figure 1:**
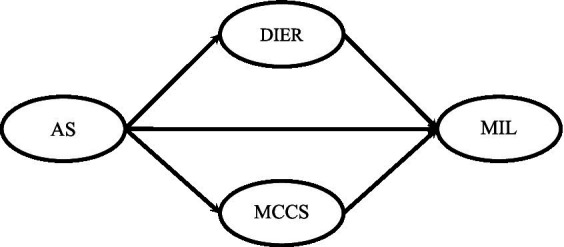
The hypothesized model of the relationship of acculturative stress with meaning in life through the mediating role of difficulty in emotion regulation and meaning-centered coping style.

Taken together, these conceptual and empirical insights highlight the need for an integrative approach that clarifies the indirect pathways linking acculturative stress to MIL. Despite existing insights into the separate associations among acculturative stress, DIER, MCCS, and MIL, the underlying mechanisms connecting these constructs in a unified explanatory model remain underexplored. What distinguishes the current study is its emphasis on a dual-mediation framework that not only identifies *what* mediates the relationship between acculturative stress and MIL but also clarifies *how* these mediators (DIER as a marker of emotional processing deficits, and MCCS as a meaning-oriented adaptive strategy) jointly operate within the broader stress-meaning system. This theoretical integration is essential because it moves the field beyond fragmented findings toward a more cohesive understanding of how international students manage cultural stress through emotional and meaning-centered pathways. Moreover, by situating the analysis within a population that is increasingly relevant in the context of global academic mobility (i.e., international students in Germany) this study contributes both theoretical depth and contextual relevance to the literature on meaning-making under cultural transition stress. Therefore, this study aims to investigate the relationship between acculturative stress and MIL, focusing on two mediating pathways of DIER and MCCS. The research question of the present study was whether acculturative stress is significantly associated with MIL among international students, and whether this relationship is mediated by DIER and MCCS. The hypotheses of the current study were (1) acculturative stress is significantly associated with MIL, (2) the relationship between acculturative stress and MIL is mediated by DIER, and (3) the relationship between acculturative stress and MIL is mediated by MCCS. Figure 1 indicates the hypothesized model of the study.

## Method

The current research employed a descriptive-correlational design through structural equation modeling (SEM).

### Participants

The target population consisted of international students enrolled in German universities in 2024. From this population, a total of 443 individuals were recruited through convenience sampling. Regarding sample adequacy, although SEM has its own guidelines, it shares similarities with multivariate regression models. According to Stevens (1996, as cited in Momeni et al., 2022), a general recommendation is to include at least 15 participants per observed variable when using ordinary least squares regression, which can reasonably extend to SEM practices (Hooman, 2014). Additionally, Loehlin (1992, as cited in Momeni et al., 2021) suggested that SEM models incorporating two to four latent constructs should include no fewer than 100–200 participants. In the present study, the hypothesized model included multiple predictors such as dimensions of acculturative stress, DIER, MCCS, and components of MIL, totaling 15 measured variables. Therefore, the minimum required sample was estimated to be 225 participants. To ensure adequacy, a power analysis using G*Power version 3.1.9.6 (Faul et al., 2007) was also conducted. Since a fully identical SEM model could not be found in existing literature, pairwise relationships between key constructs were assessed individually. The largest required sample size was determined based on the association between acculturative stress and MCCS considered in the study of Liw (2020) which indicated a required sample size of 239 cases. In this case, Pearson’s coefficient from prior literature (i.e., Liw, 2020) was utilized, and an alpha level of 0.05 and power of 0.80 were selected for the calculation. Although the initial target was to recruit 320 students, this number was raised to 384 to anticipate 20% possible attrition. Ultimately, 443 complete responses were included in the final analysis.

To be eligible for participation, students needed (1) to have resided in Germany for a minimum of 1 year, (2) be at least 18 years old, and (3) be formally enrolled in undergraduate, master’s, or doctoral programs. Individuals were excluded if (1) they were currently using psychiatric medication, (2) had experienced the loss of a loved one within the past 3 months, or (3) had been diagnosed with a severe psychological condition prior to migration, as self-reported.

The age range of participants was from 18 to 49 years, with a mean age of 26.56 years (SD = 4.65). The average ages for men and women were 26.51 (SD = 4.24) and 26.65 (SD = 4.91), respectively. The sample consisted of 168 male students (37.9%), 266 female students (60%), 6 participants (1.4%) who did not disclose their gender, and 3 individuals (0.7%) who identified as another gender. In terms of relationship status, 307 participants (69.3%) were single, 92 (20.8%) were in a committed relationship, 40 (9%) were married, 3 (0.7%) were divorced, and 1 participant (0.2%) was widowed. All students had lived in Germany for at least 1 year. The average length of stay among participants was 37.87 months (SD = 24.76), ranging from 12 to 120 months. Fifty-one participants (11.5%) reported living with a family member, while 392 (88.5%) lived independently. The sample was geographically diverse: 226 participants (51.1%) were from Asia, 100 (22.6%) from Europe, 29 (6.5%) from Africa, 26 (5.9%) from North America, 40 (9%) from South America, and 1 participant (0.2%) from Oceania.

### Instruments

#### Acculturative stress scale for international students (ASSIS-36)

To assess the levels of acculturative stress experienced by international students, the present study utilized the 36-item scale originally developed by Sandhu and Asrabadi (1994). This instrument evaluates stress related to cultural adjustment across six dimensions: perceived discrimination (e.g., *I feel that I receive unequal treatment*), homesickness (e.g., *I feel sad leaving my relatives behind*), perceived hatred (e.g., *Others do not appreciate my cultural values*), fear (e.g., *I generally keep a low profile due to fear*), stress from cultural changes (e.g., *I feel uncomfortable to adjust to new cultural values*), and guilt (e.g., *I feel guilty to leave my family and friends behind*). Items are scored on a 5-point Likert scale, ranging from one (strongly disagree) to five (strongly agree), resulting in possible total scores from 36 to 180, where higher totals denote greater acculturative stress. In the original study, the total scale yielded a Cronbach’s alpha of 0.87 (Sandhu and Asrabadi, 1994). In the current sample, internal consistency values were as follows: discrimination (*α* = 0.87), homesickness (*α* = 0.65), hatred (*α* = 0.86), fear (*α* = 0.67), cultural change (*α* = 0.45), guilt (*α* = 0.45), and overall stress (*α* = 0.93).

#### Difficulties in emotion regulation scale (DERS)

Emotion dysregulation was measured using the DERS developed by Gratz and Roemer (2004), a 36-item self-report tool. This instrument explores six specific areas: nonacceptance of emotions (e.g., *When I’m upset, I become angry with myself for feeling that way*), challenges in pursuing goals when distressed (e.g., *When I’m upset, I have difficulty concentrating*), lack of behavioral control under emotional strain (e.g., *When I’m upset, I become out of control*), low emotional awareness (e.g., *I am attentive to my feelings*), limited access to adaptive emotion regulation techniques (e.g., *When I’m upset, I start to feel very bad about myself*), and confusion about emotions (e.g., *I am confused about how I feel*). Participants responded on a scale from one (almost never) to five (almost always). Total scores range from 36 to 180, with higher values indicating more severe difficulties in emotional regulation. The original developers reported an overall alpha of 0.93 (Gratz and Roemer, 2004), while the present study found alphas of 0.95 for the total scale, and 0.91, 0.89, 0.88, 0.81, 0.90, and 0.87 for the respective subscales.

#### Meaning-centered coping scale

To evaluate coping strategies based on life meaning, the meaning-centered coping scale by Eisenbeck et al. (2022) was employed. This scale comprises nine items that reflect a unified factor related to MCCS in challenging situations. The items encompass various cognitive, emotional, and behavioral strategies such as maintaining hope, helping others, and finding purpose in adversity (e.g., *I have found a personal meaning in the current situation; I have faith that something positive will come out of this; I will get out of this situation stronger than I was before*). Participants rate each item on a 7-point Likert scale, from one (strongly disagree) to seven (strongly agree), yielding total scores between nine and 63. Higher scores denote stronger reliance on MCCS. Eisenbeck et al. (2022) reported Cronbach’s alpha values ranging from 0.81 to 0.94 across countries. In the current study, the reliability for the total scale was 0.87.

#### Meaning in life questionnaire (MLQ)

To assess participants’ presence and search of meaning in life, MLQ developed by Steger et al. (2006) was employed. The instrument comprises two distinct components, namely POM, which reflects the extent to which individuals perceive their lives as meaningful (e.g., *I understand my life’s meaning*), and SFM, which captures the active striving for deeper or more significant life meaning (e.g., *I am looking for something that makes my life feel meaningful*). MLQ consists of 10 items rated on a 7-point Likert scale, ranging from one (Absolutely untrue) to seven (Absolutely true). Each component is represented by five items, yielding two separate scores. Each component ranges from five to 35, with higher scores indicating a stronger presence or SFM, respectively. In the original validation research (Steger et al., 2006) Cronbach’s alpha coefficients were 0.86 for POM component and 0.87 for SFM component. In the present study, the internal consistency coefficients for POM and SFM, were *α* = 0.91, and *α* = 0.88, respectively.

### Procedure

Having taken necessary approvals to conduct this study from relevant authorities at the University of Siegen, Germany, and by securing the ethics code for this study from the ethics committee of the University of Siegen (code “LS_ER_20_2023”), preliminary steps of research were done. First, we wrote the study protocol and registered it on OSF.io website (REMOVED FOR PEER REVIEW). The given protocol outlined all stages of the current research process. There were six hypotheses in our preregistered protocol, among which the fourth hypothesis was analyzed in the present article. Second, the questionnaires of the study were prepared as online questionnaires. During this phase, items of each questionnaire, along with its response options, were uploaded to the limesurvey.org website. The packaging of the questionnaire was performed in the following way: demographic information from participants was obtained first, and depending on fulfillment or non-fulfillment of the eligibility criteria required to participate in the study based on their demographic information, access to the main questionnaires of the study would be provided or not. The website generated a link that participants could use to access the questionnaires page. Remarkably, participants received items in both English and German, allowing them to respond in their preferred language. Each item was presented first in English, followed by its German translation in brackets. All instruments were originally in English, which was selected as the main language due to its widespread use among international students. However, to support those more fluent in German, published German versions of the instruments were used where available. These were not translated by the researchers, and no back-translation was conducted. Instead, the German equivalent of each item was placed directly below the English item. The reported reliability coefficients (Cronbach’s alpha) are based on values from previous studies using the original English versions of the instruments. The questionnaire package consisted of 19 demographic questions, 91 main questions and three attention-check questions. A pilot trial involving some students was done to estimate how much time it took to answer the questionnaires. The minimum time a respondent would take to answer the questionnaire package was set at 20 min. Third, the questionnaires were administered to the participants. To this end, the distribution of the link to the questionnaire was enabled through the International Office at the University of Siegen for international students at Siegen and through various international offices at other German universities. Then, the link to the questionnaire was sent to the students with an accompanying email introducing the purpose of the study and assured respondents that no personal identifiable information would be collected, and data would remain confidential to the researchers. Besides, informed consent to participate was obtained at the end of the email. Students could proceed to the questionnaire page by checking an initial option indicating their consent to participate. Fourth, completed questionnaires were considered. Although informed consent was obtained from all participants at the beginning of the study, no specific information regarding the return of results or feedback to participants was provided. The study was conducted anonymously, and participants were not given access to individual feedback or study outcomes. This decision was made to ensure participant confidentiality and to maintain the integrity of the research process. In total, 668 international students participated in the study, of whom 225 were excluded from the final analysis for three reasons. One student was excluded due to having lived in Germany for less than a year, 60 students were removed for incorrectly answering all three attention-check questions, and 162 were excluded for completing the questionnaires too quickly (under 20 min), and two were excluded for prolonged response times (2 days). Ultimately, 443 participants were included in the final analysis.

### Statistical analyses

The remaining questionnaires were analyzed using Pearson’s correlation coefficient and SEM in SPSS-26 and LISREL 10.20 software, respectively. Prior to the analyses, SEM assumptions including distribution normality, error independence, and multicollinearity were examined. To examine the normality of the research variables, the skewness and kurtosis of the distribution of scores were used, the results of which showed that the distribution of scores of all variables is normal (with the range of distribution between +1.5 and −1.5). The Durbin-Watson test was used to evaluate the independence of the errors, which showed no correlation between the errors (D.W = 1.73, range between 1.5 and 2.5 is acceptable). Variance Inflation (VIF) and tolerance were used to evaluate the multicollinearity between the predictor variables. The results showed that there is no alignment between the variables (VIF amplitude less than 5 and tolerance higher than 0.1). The results of these assumptions are presented in Table 1. To determine the adequacy of the proposed model with the data, we used a combination of fitness indicators such as chi-square (χ^2^), normalized chi-square measure (chi-square ratio of degrees of freedom), good fit indices (GFI), normalized fit (NFI), adaptive fit (CFI) and root mean squared error approximation (RMSEA). To conduct SEM, in the first step, outliers were identified and removed using the Mahalanobis distance method, resulting in the exclusion of 14 participants. The analysis was then conducted on a final sample of 429 individuals. Subsequently, SEM was employed, incorporating variables in their respective roles as predictors, mediators, and criterion. The initial model demonstrated strong fit indices for most required benchmarks. Following the recommendations of LISREL for modifications, the final optimized model, which is presented below, achieved an excellent overall fit.

**Table 1:** The mean, standard deviation, and information regarding assumptions of SEM.

Variable	Min	Max	M	SD	Skewness	Kurtosis
**Acculturative stress**	26	121	62.93	16.79	0.335	−0.101
Perceived discrimination	8	39	19.79	6.65	0.409	−0.224
Homesickness	4	20	12.26	3.54	−0.206	−0.440
Perceived Hate	5	24	10.15	4.23	0.798	0.160
Fear	4	17	8.19	3.25	0.551	−0.502
Culture shock	3	15	7.83	2.47	0.256	−0.140
Guilt	2	10	4.69	2.07	0.459	−0.551
**Difficulty in emotional regulation**	36	175	91.42	26.72	0.561	−0.209
Nonacceptance of emotional responses	6	30	14.50	6.63	0.626	−0.627
Difficulty engaging in Goal-directed behavior	5	25	16.55	5.05	−0.156	−0.825
Impulse control difficulties	6	30	13.01	5.37	0.876	0.209
Lack of emotional awareness	6	29	15.35	4.91	0.219	−0.496
Limited access to emotion regulation strategies	8	40	19.89	7.72	0.615	−0.421
Lack of emotional clarity	5	25	12.10	4.41	0.593	−0.211
**Meaning-centered coping style**	9	63	47.25	9.93	−0.889	1.008
**Meaning in life**						
Presence of Meaning	5	35	23.16	7.60	−0.383	−0.631
Search for Meaning	5	35	24.93	7.02	−0.873	0.439

*n* = 429.

## Findings

### Descriptive statistics

Means, standard deviations, skewness, and kurtosis values are presented in Table 1.

### Bivariate correlations

Pearson’s correlation coefficients among acculturative stress, DIER, MCCS, POM, and SFM are reported in Table 2. The majority of the correlations were statistically significant (*p < 0*.01), indicating substantial two-way relationships among the study variables. The relationship between the length of stay in Germany and other study variables was examined using Pearson’s correlation coefficient. Results indicated a significant positive correlation between the duration of stay and two subscales of acculturative stress: discrimination (*r* = 0.139, *p* < 0.01) and perceived hate (*r* = 0.104, *p* < 0.05). Moreover, the length of stay was also significantly associated with the total acculturative stress score (*r* = 0.119, *p* < 0.05).

**Table 2:** The correlation coefficient between the variables.

Variable	1	2	3	4	5	6	7	8	9	10	11	12	13	14	15	16
**1. AS**	–															
2. PD	0.879^**^	–														
3. HS	0.600^**^	0.298^**^	–													
4. PH	0.849^**^	0.801^**^	0.301^**^	–												
5. FE	768^**^	0.624^**^	0.291^**^	0.613^**^	–											
6. CS	0.680^**^	0.450^**^	0.512^**^	0.445^**^	0.499^**^	–										
7. GU	0.501^**^	0.253^**^	0.511^**^	0.260^**^	0.307^**^	0.301^**^	–									
**8. DIER**	387^**^	0.257^**^	0.230^**^	0.265^**^	0.406^**^	0.347^**^	0.322^**^	–								
9. NER	0.354^**^	0.230^**^	0.193^**^	0.267^**^	0.370^**^	0.269^**^	0.352^**^	0.828^**^	–							
10. DEGB	0.220^**^	0.144^**^	0.191^**^	0.086	0.214^**^	0.252^**^	0.182^**^	0.690^**^	0.435^**^	–						
11. ICD	0.318^**^	0.213^**^	0.182^**^	0.221^**^	0.347^**^	0.300^**^	0.225^**^	0.809^**^	0.567^**^	0.566^**^	–					
12. LEA	0.200^**^	0.122^*^	0.087	0.190^**^	0.205^**^	0.151^**^	0.188^**^	0.567^**^	0.394^**^	0.152^**^	0.240^**^	–				
13. LAERS	0.366^**^	0.266^**^	0.201^**^	0.234^**^	0.386^**^	0.336^**^	0.285^**^	0.919^**^	0.725^**^	0.627^**^	0.766^**^	0.396^**^	–			
14. LEC	0.308^**^	0.186^**^	0.218^**^	0.214^**^	0.330^**^	0.286^**^	0.229^**^	0.798^**^	0.612^**^	0.421^**^	0.570^**^	0.565^**^	0.630^**^	–		
**15. MCC**	−0.292^**^	−0.240^**^	−0.106^*^	−0.220^**^	−0.315^**^	−0.264^**^	−0.155^**^	−0.557^**^	−0.376^**^	−0.366^**^	−0.384^**^	−0.415^**^	−0.573^**^	−0.457^**^	–	
16. POM	−0.220^**^	−0.167^**^	−0.078	−0.172^**^	−0.244^**^	−0.172^**^	−0.174^**^	−0.530^**^	−0.401^**^	−0.309^**^	−0.381^**^	−0.381^**^	−0.498^**^	−0.494^**^	0.607^**^	–
17. SFM	0.207^**^	0.138^**^	0.214^**^	0.152^**^	0.183^**^	0.124^*^	0.124^*^	0.197^**^	0.164^**^	0.164^**^	0.182^**^	−0.066	0.188^**^	0.217^**^	−0.036	−0.274^**^

*p < 0.05, **p < 0.01. AC, Acculturative Stress; CS, Culture Shock; DEGB, Difficulty Engaging in Goal-Directed Behavior; DIER, Difficulties in Emotion Regulation; FE, Fear; GU, Guilt; HS, Homesickness; ICD, Impulse Control Difficulties; LAERS, Limited Access to Emotion Regulation Strategies; LEC, Lack of Emotional Clarity; LEA, Lack of Emotional Awareness; MCCS, Meaning Centered Coping Style; NER, Nonacceptance of Emotional Responses; PD, Perceived Discrimination; PH, Perceived Hate; POM, Presence of Meaning; SFM, Search for Meaning.

The bolded labels represent overall scale scores.

### Structural equation modeling

**Figure 2:**
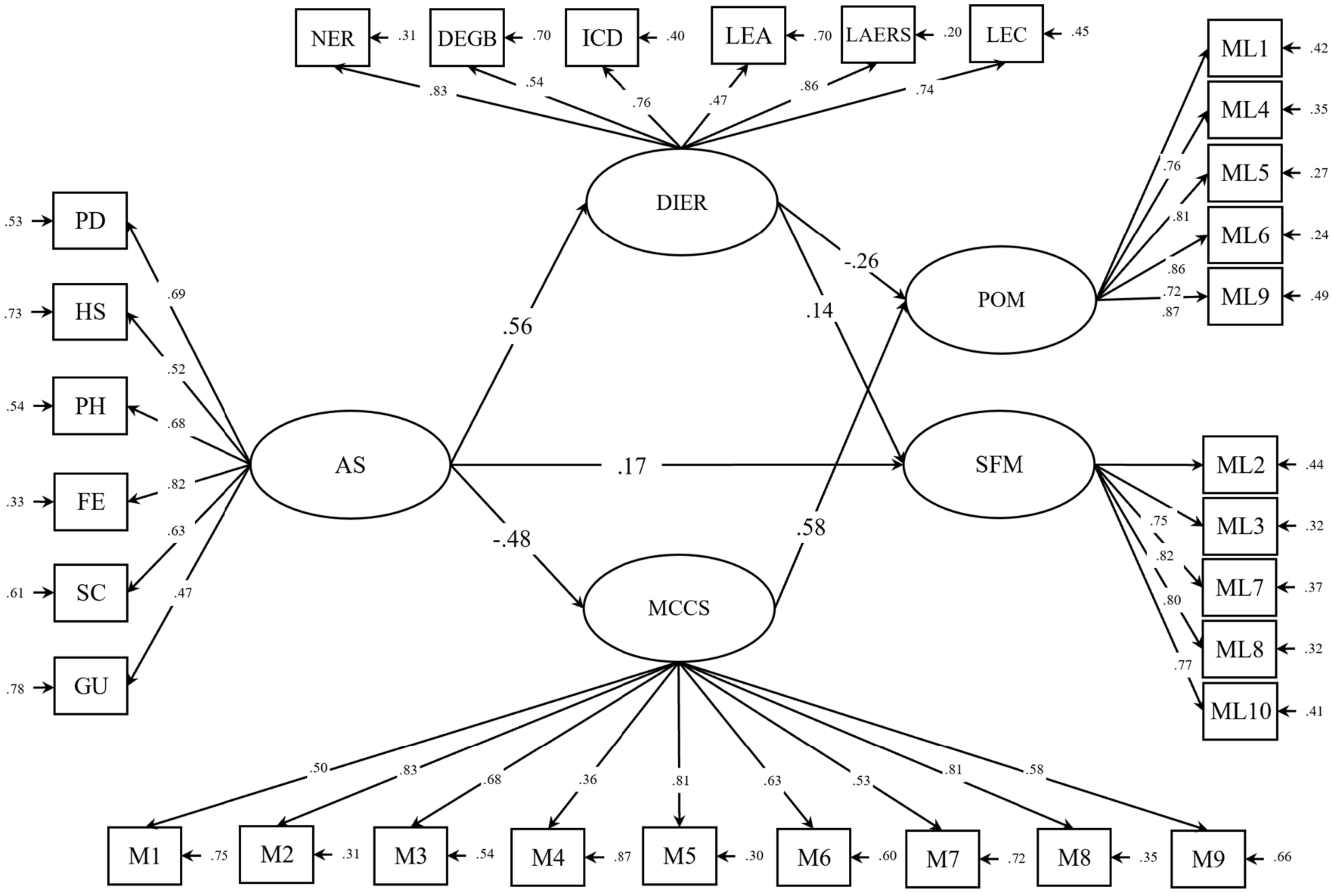
The final model of the relationship between acculturative stress and meaning in life through difficulty in emotion regulation and meaning-centered coping style; AC, Acculturative Stress; CS, Culture Shock; DEGB, Difficulty Engaging in Goal-Directed Behavior; DIER, Difficulties in Emotion Regulation; FE, Fear; GU, Guilt; HS, Homesickness; ICD, Impulse Control Difficulties; LAERS, Limited Access to Emotion Regulation Strategies; LEC, Lack of Emotional Clarity; LEA, Lack of Emotional Awareness; MCCS, Meaning Centered Coping Style; NER, Nonacceptance of Emotional Responses; PD, Perceived Discrimination; PH, Perceived Hate; POM, Presence of Meaning; SFM, Search for Meaning.

To examine the simultaneous relationships among the variables, structural equation modeling (SEM) was conducted. The model output is presented in Figure 2, and the goodness-of-fit statistics are summarized in Table 3.

**Table 3:** Fit indices for the developed model.

Model fit indices	X^2^	df	X^2^/df	GFI	AGFI	NFI	IFI	CFI	RMSEA
Obtained values	865.73	403	2.14	0.92	0.90	0.95	0.97	0.97	0.052

As shown in Table 3, the proposed model demonstrated an excellent fit: Incremental Fit Index (*IFI* = 0.97), Comparative Fit Index (*CFI* = 0.97), and Normed Fit Index (*NFI* = 0.95) all exceeded recommended thresholds. Furthermore, the Root Mean Square Error of Approximation (*RMSEA* = 0.052) indicated a good fit. Additional indices such as the Goodness of Fit Index (*GFI* = 0.92) and Adjusted Goodness of Fit Index (*AGFI* = 0.90) further supported the model’s adequacy.

### Direct effects

The direct effects among variables are presented in Table 4. All direct paths were found to be statistically significant (*p <* 0.01). However, the direct path from acculturative stress to both POM and SFM was non-significant and therefore, the corresponding paths were removed from the final model.

**Table 4:** Coefficients of the model of the relationship between AS and wellbeing through social support, DIER and MCC.

Direct path	*β*	*t*-statistic
AS → DIER	0.56	6.78^**^
AS → MCCS	−0.48	−7.12^**^
AS → SFM	0.25	2.45^**^
DIER → SFM	0.14	2.005^**^
DIER → POM	−0.26	−5.096^**^
MCCS → POM	0.58	9.65^**^

^**^p < 0.01; AC, acculturative stress; DIER, difficulties in emotion regulation; MCCS, meaning centered coping style; POM, Presence of meaning; SFM, search of meaning.

### Mediation analysis

The mediating roles of DIER and MCCS in the relationship between acculturative stress and MIL were tested using the Sobel test. The results, shown in Table 5, revealed that all tested mediation effects were significant (*p <* 0.001). Specifically, DIER and MCCS mediated the effects of acculturative stress on both POM and SFM.

**Table 5:** Sobel’s test for the mediating role of DIER and MCCS in the relationship between AC and MIL.

Variables	*p*	Sobel’s test (*z*)
AC → DIER → POM	*p* < 0.001	7.21
AC → DIER → SFM	*p* < 0.001	3.74
AC → MCCS → POM	*p* < 0.001	3.51

AC, acculturative stress; DIER, difficulties in emotion regulation; MCCS, meaning centered coping style; POM, Presence of meaning; SFM, search of meaning.

### Model summary

In sum, the final model demonstrated strong fit indices and highlighted the central mediating roles of DIER and MCCS. While no significant relationship was observed between acculturative stress and POM, a significant association was identified between acculturative stress and SFM. Furthermore, DIER was found to mediate the relationships between acculturative stress and both POM and SFM. In contrast, MCCS was involved only in the relationship between acculturative stress and POM, showing no mediating role in the association with SFM.

## Discussion

The present study aimed to examine the relationships between acculturative stress and MIL among international students, with a focus on the mediating roles of DIER and MCCS. Overall, the findings indicated that acculturative stress was related to one of the components of MIL, namely SFM, and that both DIER and MCCS served as significant mediators in these associations. Notably, DIER showed significant relationships with both components of MIL, while MCCS was only associated with POM. In the following sections, each hypothesis of the study is addressed in detail.

### Relationship between acculturative stress and MIL

The first hypothesis of this study, which proposed that acculturative stress is associated with MIL, was partially confirmed. Specifically, acculturative stress was positively related to SFM but not significantly related to POM. In other words, higher levels of acculturative stress were associated with higher levels of SFM. The finding of the current study is aligned with those of previous studies (e.g., Pan et al., 2007, 2008).

To interpret this finding, it can be mentioned that findings from Pan et al. (2007, 2008) offer important insights into how MIL may help international students cope with acculturative stress. These studies suggest that the POM functions as a psychological buffer. Students with a greater POM and coherence in life tend to experience fewer negative emotional outcomes and higher life satisfaction. This remains true even when they face stressors such as language barriers, academic pressure, cultural differences, and social isolation. The proposed mechanism is that MIL helps students interpret adverse experiences as purposeful or growth-enhancing, thereby reducing the psychological burden of acculturation stress. In contrast, students with a low POM may lack a cognitive framework through which to integrate and make sense of these challenges, leaving them more vulnerable to distress. Importantly, while these findings pertain mainly to the POM, the SFM may follow a different psychological pattern under stress. For some students, actively seeking meaning amidst acculturative stress may reflect existential uncertainty or unfulfilled psychological needs, potentially intensifying their emotional struggles.

### DIER as a mediator between acculturative stress and MIL

The second hypothesis, which proposed that the relationship between acculturative stress and MIL is mediated by DIER, was confirmed. Acculturative stress showed a positive relationship with DIER, which, in turn, was positively related to SFM and negatively related to POM. In other words, higher levels of acculturative stress were associated with higher levels of DIER, which, in turn, was related to higher SFM and lower POM. Although this specific pathway has not been directly examined in prior research, its two constituent links are aligned with previous studies. As such, acculturative stress has been found to significantly predict DIER (e.g., Awasthi et al., 2022; Cheung et al., 2020; Mayorga et al., 2018), and in turn, DIER have been associated with lower levels of MIL (e.g., Baquero-Tomás et al., 2023; Lin, 2022).

To interpret this finding, the POM is cultivated when individuals can integrate external experiences with their internal values, goals, and sense of identity (Brown et al., 2007). This process requires reflective capacity and emotional stability, both of which are undermined when emotion regulation is compromised. In contrast, the SFM, although sometimes viewed as a positive motivational construct, may in contexts of emotional distress reflect existential confusion and psychological fragmentation rather than purposeful striving. Under the psychological strain of acculturative stress, individuals often lose coherence in their self-narratives and begin to experience life as fragmented and disjointed. When these stressors co-occur with DIER, the mental space needed for meaning-making becomes increasingly unavailable. As documented by Cheung et al. (2020) and Mayorga et al. (2018), emotion dysregulation serves as a critical mediating mechanism through which acculturative stress exerts its deleterious effects on psychological outcomes.

Emotion regulation difficulties, such as poor impulse control, low distress tolerance, and an inability to identify or manage negative emotions, disrupt the very capacities that support meaning construction. Marco et al. (2021) found that emotion regulation deficits are associated with lower POM and a maladaptive SFM. In such contexts, SFM may indicate psychological disorientation rather than meaningful striving. Similarly, Lin (2022) showed that adaptive strategies such as cognitive reappraisal were positively associated with a strong sense of meaning, whereas maladaptive strategies like emotional suppression were negatively linked to both meaning dimensions.

The mediating role of DIER in this process has been supported empirically. Yi (2018) found that among Chinese international students in the U.S., acculturative stress significantly reduced the sense of MIL, which in turn predicted lower psychological wellbeing. Pan et al. (2008) similarly identified MIL as a resilience factor: migrants who retained a strong MIL experienced more positive affect despite cultural challenges. Importantly, these findings also point to a bidirectional relationship. DIER not only mediates the effects of acculturative stress on MIL, but are themselves exacerbated by the loss of existential coherence. Once the meaning-making capacity is impaired, emotional distress tends to escalate, creating a recursive cycle that further erodes psychological functioning.

### MCCS as a mediator between acculturative stress and MIL

The third hypothesis, which proposed that the relationship between acculturative stress and MIL is mediated by MCCS, was partially supported. Acculturative stress was negatively related to MCCS, which was positively related to POM but not significantly related to SFM. In other words, higher levels of acculturative stress were associated with lower levels of MCCS, and a lower level of MCCS was related to a lower level of POM. While the exact pathway explored in the current study has not been explicitly investigated in previous research, its two underlying components are supported by existing literature. Specifically, acculturative stress has been shown to significantly relate to MCCS (e.g., Folkman, 2008; Halama, 2014; Liw, 2020), and in turn, MCCS was linked to greater MIL (e.g., Liw, 2020).

To interpret this finding, it can be said that the relationship between acculturative stress and MIL can be theoretically explained through the mediating role of MCCS, drawing upon the frameworks of Folkman (2008), Frankl (1992), and Park (2010). Acculturative stress, stemming from challenges such as linguistic barriers, social exclusion, value conflicts, and emotional isolation (Berry, 1997), often disrupts one’s sense of coherence and purpose. According to Folkman’s revised stress and coping model, when individuals are exposed to uncontrollable stressors, they may shift from problem-focused or emotion-focused strategies toward MCCS. This approach involves cognitive reappraisal, redefinition of personal goals, and reinterpretation of the stressful event in a way that aligns with the individual’s values and existential beliefs. Simultaneously, Frankl’s logotherapy emphasizes the human capacity to find meaning in suffering by orienting toward a “why” that enables endurance through any “how.” In the context of international students, such coping can protect or even restore their MIL. Moreover, based on Steger et al.’s (2006) distinction between POM and SFM, it is evident that under conditions of cultural displacement and existential disorientation, students may experience a decline in the former and an activation of the latter, often as an attempt to compensate for a perceived void.

This dynamic is vividly reflected in the lived experience of international students who frequently face cumulative psychological pressures during cultural transition. The erosion of familiar support systems, identity anchors, and cultural norms may compromise their sense of continuity and significance. As POM diminishes, students are often thrust into an active SFM, often not as a sign of growth, but as a reaction to confusion or anxiety. In such instances, MCCS becomes a critical regulatory mechanism. When students are able to reframe their academic journey abroad as a meaningful endeavor (such as a step toward professional contribution, personal growth, or familial legacy) they can transform distress into a narrative of purpose. This transformation facilitates the reintegration of meaning into their lives and may gradually shift their state from SFM to POM. Therefore, MCCS does not merely mitigate distress; it enables individuals to reinterpret adversity as a context for meaning reconstruction, thereby buffering the psychological consequences of acculturative stress (Park, 2010; Rahgozar and Giménez-Llort, 2020). In sum, this study confirms the conceptual pathway whereby acculturative stress influences MIL (both in terms POM and SFM) through the mediating role of MCCS. While acculturative stress can undermine an individual’s MIL, MCCS serves as a transformative tool, enabling students to reconstruct meaning and regulate the emotional consequences of cultural disruption.

### Implications of this study

The present study offers valuable implications at the research, social, and therapeutic levels. At the research level, the findings contribute to a more nuanced understanding of how acculturative stress relates to MIL among international students, highlighting the mediating roles of DIER and MCCS. These results underline the importance of meaning-making and emotional processing in adapting to cross-cultural transitions. Future research may benefit from further exploring these mediators in different populations and contexts, thereby enhancing theoretical frameworks concerning psychological adaptation and existential wellbeing in multicultural environments.

At the social level, this study underscores the importance of developing university and community-based programs that help international students find meaning in their experiences. Institutions can support these students by offering structured programs that include reflective workshops, group discussions on personal values and cultural integration, and training in adaptive coping mechanisms. Such initiatives may help reduce the psychological burden of acculturative stress and promote a stronger MIL, thereby improving students’ overall academic and social functioning. Furthermore, public policies that support the psychological adjustment of international students, through accessible mental health services and culturally sensitive interventions, can foster more inclusive and supportive academic environments.

An additional recommendation is for universities to establish dedicated counseling centers or services specifically designed for international students, where at least some counselors share the students’ cultural background or speak their native language. This approach can significantly improve the therapeutic alliance, as students may feel more understood, respected, and comfortable discussing culturally sensitive topics. Language barriers, cultural stigma around mental health, and unfamiliarity with the host country’s support systems often prevent international students from seeking help. Having access to counselors who understand their cultural values and emotional expressions can lower these barriers and encourage early intervention. Furthermore, culturally aligned counseling can serve not only as a space for psychological support but also as a bridge for academic and social adaptation, ultimately promoting integration and wellbeing within the host society.

At the therapeutic level, the findings suggest that clinicians and counselors working with international students should pay particular attention to issues surrounding MIL and emotion regulation. Integrating meaning-centered coping techniques into therapeutic interventions may help students better navigate the challenges of acculturative stress while fostering a deeper MIL and psychological resilience. In this context, Acceptance and Commitment Therapy (ACT) emerges as a promising approach, given its emphasis on value-based action, emotional acceptance, and mindfulness. ACT can be particularly helpful in assisting students to acknowledge and regulate difficult emotions while reconnecting with their core values and life goals, which may ultimately strengthen their MIL (Steger et al., 2013).

### Limitations of the current study

This study has several limitations that should be acknowledged. First, the use of self-report measures may introduce bias, as participants’ responses can be influenced by social desirability or inaccurate self-perceptions. While self-reports offer valuable insight into internal psychological processes, they may limit the ecological validity of the findings. Moreover, although SEM allowed for the examination of complex relationships among variables, the design of the study was cross-sectional, which precludes any conclusions about causality. The observed associations, therefore, should be interpreted as correlational. Another limitation lies in the sample selection, as the study focused solely on international students residing in Germany. Cultural, educational, and legal environments differ across countries, meaning the stressors and coping mechanisms identified here may not fully reflect those experienced by international students elsewhere. As such, caution is needed when attempting to generalize the results beyond this specific context. Another limitation concerns the use of validated instruments that lacked formal cultural or linguistic adaptation for the target population, which may have affected participants’ understanding of some items and contributed to lower engagement in certain cases. However, the application of attention checks and the analysis of average response times helped to reduce the potential impact of this issue. Moreover, the internal consistency of the Cultural Change (*α* = 0.45) and Guilt (*α* = 0.45) subscales of the Acculturative Stress Scale for International Students was relatively low. However, these subscales demonstrated strong factor loading in the initial confirmatory factor analysis, indicating a good conceptual alignment with the broader construct of acculturative stress. These findings may reflect cultural nuances in the interpretation of the items, translation-related issues, or limitations inherent in the brevity of the short-form scale. Therefore, interpretations involving this subscale should be made with caution, and future studies are encouraged to further investigate its psychometric performance in cross-cultural contexts.

### Recommendations for future research

To address these limitations, future research should consider employing mixed-method approaches, combining qualitative interviews with quantitative assessments to gain a deeper understanding of the studied constructs. Using more objective or multi-informant data sources could also strengthen the reliability of findings. Furthermore, longitudinal designs are recommended to explore how acculturative stress, MIL, and DIER evolve over time, and to better infer causality. Replicating this research in diverse cultural and national contexts would also be essential for identifying universal versus culture-specific patterns, thereby enhancing the generalizability and impact of the findings.

## Data Availability

The datasets presented in this study can be found in online repositories. The names of the repository/repositories and accession number(s) can be found at: https://osf.io/prfg5/?view_only=2bbdee61cb1844a08dfad5dcc6f031da.
